# Use of the KDQOL-36™ for assessment of health-related quality of life among dialysis patients in the United States

**DOI:** 10.1186/s12882-019-1295-0

**Published:** 2019-04-01

**Authors:** Dena E. Cohen, Andrew Lee, Scott Sibbel, Deborah Benner, Steven M. Brunelli, Francesca Tentori

**Affiliations:** 1 DaVita Clinical Research, 825 S 8th St, Minneapolis, MN 55404 USA; 2 DaVita, Inc., Denver, CO USA

**Keywords:** Quality of life, ESRD, Dialysis

## Abstract

**Background:**

Health-related quality of life (HRQOL) is a key outcome for dialysis patients, and its assessment is mandated by the Centers for Medicaid and Medicare Services. The Kidney Disease Quality of Life (KDQOL-36™) survey is widely used for this assessment. KDQOL-36™ completion rates, and the distributions of scores and item responses, have not been examined in a large, nationally representative cohort of dialysis patients.

**Methods:**

This retrospective, observational study considered 413,951 survey opportunities contributed by adult patients who received dialysis at a large dialysis organization in the United States during calendar years 2014, 2015, and 2016 and were not Veterans Affairs beneficiaries.

**Results:**

During the study period, 240,343 unique patients completed a total of 330,412 surveys (overall completion rate 79.8%). Mean domain scores on the physical component summary (PCS), mental component summary (MCS), burden of kidney disease (BKD), symptoms and problems of kidney disease (SPKD), and effects of kidney disease (EKD) subscales were 36.6, 49.0, 51.3, 78.1, and 73.0, respectively. Scores were similar across dialysis modalities. Patient perceptions of general health were not correlated (R < 0.05) with PCS or SPKD. The SPKD showed ceiling effects: among patients treated with in-center hemodialysis, for all 12 items, < 10% of patients were “extremely bothered,” while > 65% of patients reported being “not at all” or only “somewhat bothered;” for 3 items, > 85% of patients gave these latter two responses. Interdialytic weight gain was not correlated with patient-reported shortness of breath, PCS, or SPKD.

**Conclusions:**

Survey completion rates for the KDQOL-36™ were high, and scores were similar across dialysis modalities. Ceiling effects were observed for SPKD. Revision of the KDQOL-36™ to address factors that are most important to contemporary dialysis patients may be warranted.

**Electronic supplementary material:**

The online version of this article (10.1186/s12882-019-1295-0) contains supplementary material, which is available to authorized users.

## Background

Health-related quality of life (HRQOL) is a critically important outcome for patients with end-stage renal disease (ESRD). In recognition of this, in 2008, the Centers for Medicare and Medicaid Services (CMS) mandated annual assessment of HRQOL as part of its conditions for coverage for ESRD facilities [1]. The National Quality Forum selected the Kidney Disease Quality of Life Short-Form survey (KDQOL™-36) as the tool of choice for assessing this outcome in adult patients with ESRD; assessment is required within 4 months of initiating dialysis, and annually thereafter [2]. This 36-question survey instrument was published in 2000 [3], based upon a longer KDQOL instrument first developed in 1994 [4]. The KDQOL™-36 contains 5 subscales: the Physical Component Summary (PCS), Mental Component Summary (MCS), Burden of Kidney Disease (BKD), Symptoms and Problems of Kidney Disease (SPKD), and Effects of Kidney Disease (EKD). The first 2 subscales are a generic measure of HRQOL (and are identical to the SF-12), whereas the last 3 assess issues specific to patients with ESRD or earlier stages of chronic kidney disease.

In spite of the importance of HRQOL as an outcome, and the long-standing use of the KDQOL™-36 for its assessment among patients with ESRD, validation of the instrument has been fairly limited [5]. Indeed, the renal community in the United States has expressed concern regarding the relevance of the KDQOL™-36 in the current environment [6], given changes in ESRD care that have occurred since its development. Recent work has identified factors beyond those included in the KDQOL™-36 that patients have identified as important to their quality of life [7, 8], raising questions as to whether a revision of the KDQOL™-36 may be necessary.

Here, we sought to understand the survey response rates, distribution of component scores, and distribution of responses to individual items among a large, nationally representative contemporary cohort of US patients with ESRD. We also sought to examine correlations between component scores, individual item responses, and fluid status. In particular, given the importance of disease symptoms as a driver of HRQOL [9–12], we sought to determine the extent to which the items comprising the SPKD subscale have remained relevant since the instrument’s development.

## Methods

### Patients, timeframe, and data sources

This study considered KDQOL™-36 survey opportunities (ie, recorded instances at which the KDQOL-36™ survey was offered to a patient) that occurred between 01 January 2014 and 21 December 2016 at a large dialysis organization (LDO) operating in the United States. The LDO has clinics in 45 of the 50 states (the exceptions are Alaska, Delaware, Indiana, Vermont, and Wyoming). We considered patients who, as of the date of the survey opportunity, were ≥ 18 years of age, were not Veterans Affairs beneficiaries (contractual stipulation), and were receiving dialysis care at the LDO. All data, including KDQOL™-36 item responses, patient demographic information, and treatment information, were derived from electronic records maintained by the LDO.

Among patients treated with in-center hemodialysis (ICHD), information about treatment attendance and inter-dialytic weight gain (IDWG) in the 30 days prior to the survey opportunity was likewise derived from electronic health records. Treatment attendance records indicate whether a patient attended treatment, missed treatment due to hospitalization, or missed treatment due to other reasons (referred to as “absences.”). IDWG was considered as either the average percent of target weight gained prior to each attended treatment, or as frequent excessive IDWG based on the internal definition utilized by the LDO (> 5% of target weight in > 10% of treatment intervals).

This study was conducted using deidentified patient data; therefore, according to title 45, part 46 of the US Department of Health and Human Services’ Code of Federal Regulations, this study was deemed exempt from institutional review board (IRB) or ethics committee approval (Quorum IRB, Seattle, WA). We adhered to the Declaration of Helsinki and informed consent was not required.

### Surveys

At the LDO, KDQOL™-36 surveys are offered to patients within 4 months of their first treatment at the LDO, and a minimum of annually thereafter, in accordance with CMS policy [2]. Surveys were considered as “completed” if, at minimum, questions 1–12 (comprising the PCS and MCS) were answered. Surveys were considered as “declined” if the patient declined to complete the survey when offered (patients were not required to provide a reason for declining the survey), or if questions 1–12 were not completed. No imputation methods were used to handle missing values. All survey scores (from completed surveys and surveys with missing values in items 13–36) were calculated according to the procedures set forth by the Rand Corporation, which developed the survey. Because CMS guidelines indicate that a survey with responses to items 1–12 be regarded as complete irrespective of the status of the other items, we applied the same criteria in our analysis. Survey opportunities at which the survey was not offered to a patient for reasons of dementia, active psychosis, or other barriers to survey completion, were excluded from the analysis. On occasion, social workers will administer an off-cycle KDQOL™-36 survey in response to changes in patient circumstance (eg, severe illness, death of a caregiver, etc), resulting in more than one recorded KDQOL™-36 survey opportunity during a survey period (ie at approximately 4, 16, or 28 months of dialysis initiation). In such instances, only the first opportunity in the survey period was considered in this analysis. Responses to individual items on the KDQOL™-36 survey were used to calculate scores on the 5 subscales according to formulae specified by the developer of the instrument [13]. These formulae are specified such that a higher numeric value of score is always reflective of a better perceived quality of life. In the case of the BKD score, a higher score is reflective of a *lower* perceived burden of kidney disease.

### Statistical analyses

Data are presented as means and standard deviations, medians and interquartile ranges, or counts and proportions, as dictated by data type. Correlations between item responses, domain scores, and IDWG were calculated using Pearson correlations [14] computed with pairwise complete observations.

## Results

### Patient characteristics

This study considered a total of 413,964 survey opportunities offered to 240,343 unique patients (Additional file 1: Table S1), corresponding to a mean of 1.5 survey opportunities (standard deviation 0.7) per patient. The characteristics of the overall study cohort were similar to the national contemporary ESRD population (as documented by the United States Renal Data System) in terms of age, sex, race/ethnicity, and distribution of modalities [15] (Additional file 1: Table S2). Of the surveys offered to patients, 73,889 surveys were declined outright, with a further 9663 being completed but with missing data for at least one of items 1–12 and thus being considered as declined, for a total of 83,552 declined surveys (Additional file 1: Figure S1). The remaining 330,412 surveys, which had complete information for items 1–12, were considered as completed.

Compared to patients who declined surveys, those who completed surveys were, on average, younger, of newer dialysis vintage, had higher body mass index (BMI), and had lower Charlson Comorbidity Index (CCI) scores (Table 1). When patients were grouped by dialysis modality, those treated with in-center hemodialysis (ICHD) were generally older, more frequently diabetic, and had higher CCI scores compared to those treated with peritoneal dialysis (PD, Additional file 1: Tables S3 and S4). Within each of these modalities, patterns in demographics and clinical characteristics among patients who completed vs. declined surveys were similar to those observed in the overall cohort (Additional file 1: Tables S5 and S6).

**Table 1:** Patient demographics and clinical characteristics by KDQOL-36™ completion status

	Overall *N* = 413,964	Declined *N* = 83,552	Completed *N* = 330,412
Age, years, mean ± SD	61.4 ± 14.9	64.0 ± 15.2	60.7 ± 14.7
Sex, female, n (%)	183,613 (44.4)	36,323 (43.5)	147,290 (44.6)
Race, n (%)			
White	160,451 (38.8)	30,542 (36.6)	129,909 (39.3)
Black	145,729 (35.2)	27,588 (33.0)	118,141 (35.8)
Hispanic	72,769 (17.6)	14,858 (17.8)	57,911 (17.5)
Asian	17,445 (4.2)	5972 (7.1)	11,473 (3.5)
Other/unknown	17,557 (4.2)	4589 (5.5)	12,968 (3.9)
Modality, n (%)			
In-center hemodialysis	358,475 (86.6)	75,580 (90.5)	282,895 (85.6)
Peritoneal dialysis	45,903 (11.1)	6395 (7.7)	39,508 (12.0)
Home hemodialysis	6596 (1.6)	1058 (1.3)	5538 (1.7)
Nocturnal dialysis	2977 (0.7)	516 (0.6)	2461 (0.9)
Dialysis vintage, months, median [p25, p75]	29 [12, 57]	35 [15, 64]	28 [11, 56]
BMI, kg/m^2^, mean ± SD	28.6 ± 7.4	27.1 ± 7.0	28.9 ± 7.4
CCI, median [p25, p75]	5 [4, 7]	6 [4, 7]	5 [4, 7]
Diabetes, n (%)	283,744 (68.5)	58,449 (70.1)	225,295 (68.2)

Abbreviations: *BMI* body mass index, *CCI* Charlson comorbidity index, *SD* standard deviation

### Survey completion rates

Overall, the survey completion rate in the study population was 79.8%, with 20.2% of surveys declined (Table 2). Stratification of the population by age revealed that younger age (18–64 years) was associated with a markedly higher survey completion rate than older age (≥65 years), with completion rates of 81.8–83.8% and 76.3% respectively. Asian race was associated with a much lower completion rate (65.8%) than other racial and ethnic backgrounds. With respect to modality, PD was associated with a higher survey completion rate (86.1%) than ICHD (78.9%). Longer vintage (≥36 months) correlated with a lower completion rate (77.2%) than shorter vintage. Lower BMI or higher CCI were likewise associated with lower completion rates than higher BMI or lower CCI. When patients treated with ICHD or PD were considered separately, essentially similar trends were observed within each group as were observed for the cohort as a whole (Additional file 1: Tables S5 and S6).

**Table 2:** KDQOL-36™ survey completion by patient characteristics

	Survey Status	
	Declined	Completed
All	83,552 (20.2)	330,402 (79.8)
Age, years		
18–24	699 (16.6)	3514 (83.4)
25–34	3051 (17.7)	14,222 (82.3)
35–44	5760 (16.2)	29,883 (83.8)
45–54	11,568 (16.8)	57,283 (83.2)
55–64	19,123 (18.2)	85,723 (81.8)
65+	43,351 (23.7)	139,787 (76.3)
Sex		
Male	47,226 (20.5)	183,116 (79.5)
Female	36,326 (19.8)	147,296 (80.2)
Race/Ethnicity		
White	30,543 (19.0)	129,915 (81.0)
Black	27,588 (18.9)	118,143 (81.1)
Hispanic	14,859 (20.4)	57,913 (79.6)
Asian	5973 (34.2)	11,473 (65.8)
Other/Unknown/Missing	4589 (26.1)	12,968 (73.9)
Modality		
In-center hemodialysis	75,580 (21.1)	282,895 (78.9)
Peritoneal dialysis	6395 (13.9)	39,508 (86.1)
Home hemodialysis	1058 (16.0)	5538 (84.0)
Nocturnal dialysis	516 (17.3)	2461 (82.7)
Vintage, months		
< 12	19,210 (17.5)	90,260 (82.5)
12–24	13,020 (18.2)	58,392 (81.8)
25–36	11,055 (19.6)	45,229 (80.4)
36+	40,267 (22.8)	136,531 (77.2)
BMI		
< 18.5	5240 (30.4)	11,996 (69.6)
18.5–24	32,054 (24.3)	100,121 (75.7)
25–29	23,404 (19.7)	95,541 (80.3)
30+	22,854 (15.7)	122,754 (84.3)
CCI		
< 3	5438 (16.6)	27,404 (83.4)
3–4	17,018 (16.8)	84,515 (83.2)
5–6	32,143 (19.4)	133,309 (80.6)
7+	28,953 (25.4)	85,184 (74.6)

Abbreviations: *BMI* body mass index, *CCI* Charlson comorbidity index

### Survey scores

The five component scores of the KDQOL™-36 were calculated based on individual item responses within each completed survey. Among completed surveys (ie, those in which at minimum items 1–12 were completed, *N* = 330,412), the majority (*n* = 189,174; 57.3%) were contributed by patients who completed only one survey during the study period. The remainder were contributed by patients who completed 2, 3, or 4 surveys (30.8, 11.4, and 0.5%, respectively). The vast majority of completed surveys (88.2%) included responses to all 36 items. Responses to each of items 13–36 were missing in approximately 1% of surveys, with the exceptions of items 28 and 35 (Additional file 1: Table S7). Item 28 (Problems with your access/catheter site) was unanswered in 3.3% of surveys, and item 35 (Your sex life) was unanswered in 5.7% of surveys.

Overall, mean scores on the PCS, MCS, BKD, SPKD, and EKD were 36.6, 49.0, 51.3, 78.1, and 73.0, respectively. When surveys were stratified by either calendar year (2014, 2015, and 2016) or timing of survey with respect to vintage at the LDO (4 months, 16 months, 28 months), mean scores for each of the 5 subscales differed by ≤2 points across categories (Additional file 1: Table S8). Surveys were therefore aggregated across calendar years and vintage at the LDO.

Stratification of surveys by age category (Table 3) revealed that younger age correlated with substantially higher mean PCS (43.4 for age 18–24, 36.2 for age ≥ 65). Scores on the other 4 subscales were not substantially different across age categories, although in general, younger age categories tended to have higher mean scores than older ones. Stratification of patients by total dialysis vintage revealed a trend towards slightly higher BKD score with increasing vintage (49.2 for < 12 months on dialysis, versus 52.7 for ≥36 months); no trends were observed across the other 4 subscales. Stratification by CCI demonstrated that scores of < 3 corresponded to a mean PCS score of 41.1, compared to a mean of 34.4 for CCI ≥7. No notable differences were observed with respect to CCI category for the other KDQOL-36™ subscales (differences between categories of ≤3 points).

**Table 3:** KDQOL-36™ domain scores by patient characteristics

	PCS	MCS	BKD	SPKD	EKD
Overall	36.6 ± 12.2	49.0 ± 13.4	51.3 ± 29.8	78.1 ± 16.7	73.0 ± 22.7
Age, years					
18–24	43.4 ± 11.5	48.8 ± 12.6	49.8 ± 28.5	80.3 ± 17.0	72.9 ± 22.2
25–34	40.9 ± 11.9	48.1 ± 12.7	48.7 ± 29.0	77.9 ± 17.2	70.3 ± 23.2
35–44	39.2 ± 12.1	48.3 ± 12.9	49.6 ± 29.5	77.3 ± 17.2	70.0 ± 23.5
45–64	37.4 ± 12.1	48.1 ± 13.2	49.3 ± 29.8	76.5 ± 17.4	69.9 ± 23.6
65+	36.2 ± 12.1	48.5 ± 13.4	50.7 ± 29.9	77.3 ± 17.0	71.6 ± 23.0
Sex					
Female	35.4 ± 12.0	48.8 ± 13.6	52.2 ± 30.3	76.7 ± 16.9	73.6 ± 22.4
Male	37.5 ± 12.3	49.2 ± 13.3	50.5 ± 29.5	79.3 ± 16.5	72.5 ± 22.9
Race, n (%)					
White	34.8 ± 11.9	49.4 ± 13.3	50.0 ± 28.9	77.6 ± 16.1	72.1 ± 22.0
Black	37.9 ± 12.1	49.8 ± 13.2	55.8 ± 30.0	78.9 ± 16.7	75.7 ± 22.2
Hispanic	37.6 ± 12.6	46.9 ± 14.1	47.1 ± 30.7	77.8 ± 17.6	70.2 ± 24.0
Asian	37.7 ± 12.2	48.2 ± 13.3	44.1 ± 29.2	76.9 ± 18.4	69.4 ± 23.8
Other/unknown	37.2 ± 12.1	48.9 ± 13.3	48.7 ± 29.7	77.4 ± 17.9	72.0 ± 23.5
Charlson Comorbidity Index					
< 3	41.4 ± 11.9	48.7 ± 12.7	51.2 ± 29.3	79.3 ± 16.7	71.8 ± 22.9
3–4	38.2 ± 12.2	48.5 ± 13.1	51.0 ± 29.7	77.8 ± 17.0	71.4 ± 23.1
5–6	36.0 ± 12.1	49.1 ± 13.5	51.1 ± 29.9	78.0 ± 16.7	73.0 ± 22.6
7+	34.4 ± 11.9	49.5 ± 13.8	51.7 ± 30.0	78.2 ± 16.6	74.8 ± 22.1
Dialysis modality					
ICHD	36.3 ± 12.2	49.0 ± 13.5	50.7 ± 30.0	77.9 ± 16.9	72.6 ± 22.9
PD	38.1 ± 12.4	49.3 ± 13.1	55.7 ± 28.7	79.7 ± 15.7	75.5 ± 20.6
HHD	37.2 ± 12.6	49.3 ± 13.0	47.5 ± 28.9	78.9 ± 15.7	71.8 ± 22.0
NOC	38.1 ± 12.8	49.8 ± 13.1	54.0 ± 28.9	78.1 ± 16.0	70.1 ± 22.8
Dialysis vintage, months					
< 12	36.2 ± 12.2	48.7 ± 13.6	49.2 ± 29.3	78.2 ± 16.6	72.4 ± 22.5
12- < 24	36.9 ± 12.2	49.2 ± 13.3	51.0 ± 29.6	78.2 ± 16.7	73.2 ± 22.4
24- < 36	36.8 ± 12.2	49.1 ± 13.3	51.3 ± 29.9	78.2 ± 16.8	73.1 ± 22.6
≥ 36	36.6 ± 12.2	49.1 ± 13.4	52.7 ± 30.2	78.0 ± 16.9	73.2 ± 22.9

Abbreviations: *BKD* burden of kidney disease, *EKD* effects of kidney disease, *HHD* home hemodialysis, *ICHD* in-center hemodialysis, *MCS* mental component score, *NOC* nocturnal dialysis, *PCS* physical component score, *PD* peritoneal dialysis, *SPKD* symptoms and problems of kidney disease

With respect to race and ethnicity (Table 3), white race was associated with a lower mean score on the PCS (34.8) than other races (37.2–37.9). A substantial range in mean BKD was observed with respect to race, with black race being associated with the highest mean score (55.8), Asian race with the lowest (44.1), and other races falling in between (47.1–50.0). A similar pattern was observed with respect to EKD: Asian race corresponded to a mean score of 69.4, black race to a mean score of 75.7, and other races to intermediate mean scores (70.2–72.1). In contrast, mean SPKD scores were similar across race categories (76.9–78.9). No meaningful differences were observed between males and females in any of the 5 subscales (differences in mean score for each were < 3 points).

**Fig. 1:**
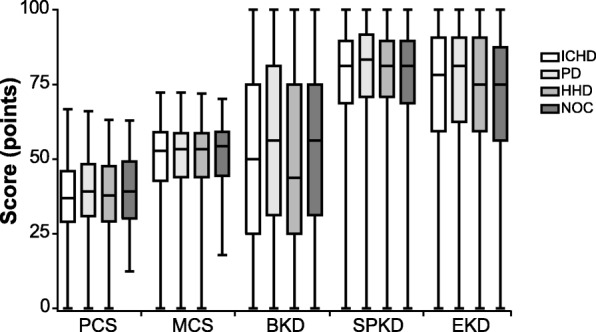
Distribution of KDQOL-36™ Domain Scores by Dialysis Modality. Minimum, lower quartile, median, upper quartile, and maximum scores are depicted for each of the five KDQOL-36™ domain scores among patients treated with each of four dialysis modalities. Abbreviations: BKD, burden of kidney disease; EKD, effects of kidney disease; HHD, home hemodialysis; ICHD, in-center hemodialysis; MCS, mental component score; NOC, nocturnal in-center hemodialysis; PCS, physical component score; PD, peritoneal dialysis; SPKD, symptoms and problems of kidney disease

Despite the demographic differences between patients treated with ICHD vs PD, scores on the 5 domains of the KDQOL-36™ were similar across dialysis modalities (Table 3 and Fig. 1). Mean scores for PCS, MS, and SPKD differed by ≤2.5 points across all 4 modalities considered. Larger differences were observed with respect to BKD and EKD scores: median BKD was 55.7 for PD, compared to 50.7 for ICHD and 47.5 for home hemodialysis (HHD). PD was associated with a median EKD of 75.5, compared to 72.6 for ICHD; lower scores were observed for nocturnal in-center hemodialysis (NOC) and HHD.

Among patients treated with ICHD, surveys were further stratified by the number of treatments missed in the 30 days preceding the survey date (Additional file 1: Table S9); missed treatments were ascribed to hospitalization or to other reasons (“absences”) based on treatment records. Compared to no missed treatments due to hospitalization in the 30 days prior to the survey date, 3 or more missed treatments due to hospitalization was associated with notably lower scores in all 5 subscales, with differences ranging from − 2.6 points (EKD) to − 5.2 points (PCS). Similarly, compared to no absences in the 30 days prior to survey date 3 or more absences correlated with lower mean scores in MCS, BKD, and SPKD (differences of − 2.5, − 6.4, and − 4.1, respectively). Differences in PCS and EKD were < 2 points between these two groups.

### Assessment of symptoms by the KDQOL-36™

**Fig. 2:**
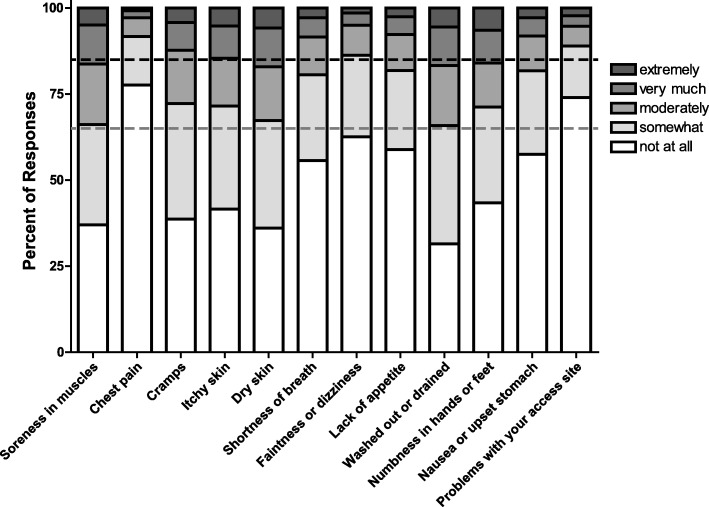
Responses to Items on the KDQOL-36™ Symptoms and Problems of Kidney Disease Subscale among Patients on In-Center Hemodialysis. Responses to the 12 items comprising the Symptoms and Problems of Kidney Disease subscale among patients treated with in-center hemodialysis who responded to each item are depicted. The question stem for all 12 items is, “During the past 4 weeks, to what extent were you bothered by each of the following?” Possible responses are not at all, somewhat, moderately, very much, and extremely. Dashed grey line indicates cumulative 65% of responses; dashed black line indicates cumulative 85% of responses

Across the study cohort, the SPKD had the highest mean score (78.1) of the 5 subscales on the KDQOL-36™. Examination of responses to the items comprising the SPKD among patients treated with ICHD demonstrated that ≥50% of patients reported being “not at all bothered” by 6 of the 12 items surveyed (items 18, 22, 23, 24, 27, and 28). Less than 10% of patients indicated being “extremely bothered” by each of the 12 items. For all 12 items, > 65% of patients indicated that they were either “not at all bothered” or only “somewhat bothered” by the symptom being queried (Fig. 2). For items 18, 22, and 28, > 85% of patients who completed the survey gave one of these two responses. These trends were even more pronounced among patients treated with PD (Additional file 1: Figure S2).

**Fig. 3:**
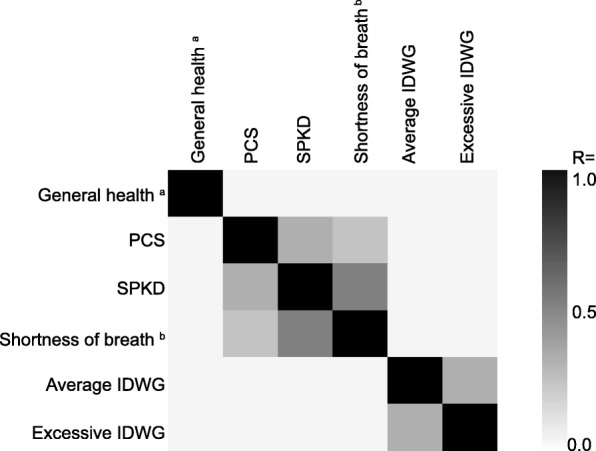
Correlation between Selected KDQOL-36™ Domain Scores, Individual Items, and Indicators of Fluid Overload among Patients on In-Center Hemodialysis. Pearson correlations between the indicated constructs among patients treated with in-center hemodialysis are shown. Average IDWG was considered as a percentage of body weight with respect to treatments in the 30 days prior to survey date. Excessive IDWG was considered as a gain of > 5% of target weight in > 10% of treatments occurring in the 30 days prior to the survey date. ^a^ Item 1: “In general, would you say your health is:” Possible responses are “excellent,” “very good,” “good,” “fair,” and “poor.”. ^b^ Item 22: “During the past 4 weeks, to what extent were you bothered by each of the following?” Possible responses are “not at all bothered,” “somewhat bothered,” “moderately bothered,” “very much bothered,” and “extremely bothered.”. Abbreviations: IDWG, interdialytic weight gain; PCS, physical component summary; SPKD, symptoms and problems of kidney disease

Pearson correlations were used to assess relationships between PCS and SPKD, as these two components both measure patient-reported physical health. PCS and SPKD were moderately correlated among patients treated with ICHD (R = 0.41, Fig. 3). However, patients’ responses to item 1 on the KDQOL-36™ (“In general, would you say your health is:” a component of PCS) did not correlate with either PCS or SPKD (R < 0.05). Neither SPKD generally, nor responses to item 22 (“Shortness of breath”), correlated with IDWG (R < 0.05). Similar results were observed among patients treated with PD (Additional file 1: Figure S3).

## Discussion

This study examined KDQOL-36™ survey response rates and scores from a large, nationally representative cohort of contemporary ESRD patients treated with four different dialysis modalities. The overall survey response rate was 78.2%, and mean component scores were similar to those reported for other US dialysis cohorts [5, 16]. Stratification of the study cohort on the basis of demographic and clinical characteristics revealed specific characteristics that were associated with lower or higher participation rates in the survey. Similarly, this stratification enabled identification of characteristics associated with particularly high or low mean scores on specific KDQOL-36™ domains. Beyond specific clinical, social, or other factors that may influence quality of life, differences between patient subgroups may be driven by differences in patient expectations with regard to their quality of life. The same set of circumstances may thus correspond to a low or high perceived quality of life, depending on the particular patient.

Notably, despite differences in patient demographics, KDQOL-36™ scores did not vary substantially across the four dialysis modalities examined. Indeed, a majority of the differences observed across modalities were less than 3–5 points, which is generally considered the threshold for minimally important differences (MID) in score on the KDQOL-36™ instrument [17, 18]. Several previous studies have examined correlations between dialysis modality and HRQOL, generally focusing on the comparison between ICHD and PD in cohorts substantially smaller than the one examined here [19–23]. Although differing to some extent with respect to particular details, these earlier studies and the findings presented here support the conclusion that HRQOL tends to be quite similar across ICHD and PD, a finding that the present study now extends to NOC and HHD as well. The consistent observation that similar HRQOL is achieved across treatment modalities, despite the fact that patients on ICHD tend to be older and sicker than those treated with other modalities, suggests that appropriate matching of individual patients to the dialysis modality that best fits their needs, rather than the modality itself, may be a key determinant of HRQOL.

This study has identified some important limitations of the SPKD subscale of the KDQOL-36™. Mean scores on the SPKD were numerically higher than mean scores on the other 4 subscales. Although SPKD was moderately correlated with PCS, as might be expected for two scores that measure physical aspects of health, mean score on the SPKD exceeded mean score on the PCS by approximately 40 points. Thus, the two scores convey very different messages about patient health: a PCS score in the 30’s is suggestive of extremely poor overall health, whereas an SPKD score of 70 or higher suggests a relatively low symptom burden. This pattern is suggestive, although not proof positive, that the SPKD subscale may be topped out.

In support of the idea that SPKD may be topped out, a majority (≥65%) of patients in the contemporary cohort studied here were “not at all” or only “somewhat” bothered by each of the symptoms queried by the instrument. Notably, this was true for 8 of these 12 items at the time that the KDQOL was first developed [4], suggesting that even within the clinical context at that time, these symptoms may not have been particularly troubling to a majority of patients. Based on the findings presented here, it appears that the 4 symptoms that were more bothersome at the time that the instrument was developed (dry skin, itchy skin, washed out or drained, and muscle soreness) are less so in the current clinical context, given the substantial improvements in dialysis delivery and other aspects of ESRD management have occurred over the past two decades. This raises the possibility that the list of symptoms queried in the SPKD may need to be refreshed to account for corresponding changes in the experience of patients on dialysis over that time.

The degree to which symptoms queried on the SPKD correlate with objective measures of patient health during the 30 days prior to survey has not been extensively examined. Here, we found that there was no correlation between fluid status (ie IDWG) in the 30 days prior to survey and a symptom that is largely driven by fluid overload, namely shortness of breath. The absence of such a correlation raises questions as to how to interpret and address patient perceptions of the symptoms reported on the KDQOL-36™, and further underscores the need to reexamine this subscale.

Strikingly, the response to item 1 on the KDQOL-36™ (“In general, would you say your health is”) was not correlated with any of the 5 subscale scores, nor with the response to any individual item on SPKD. This is notable in that patient-reported general health is thought to reflect aspects of health that are difficult to capture via clinical measures, and is independently associated with mortality risk [24]. This finding suggests that efforts to identify factors that influence perceptions of general health among dialysis patients, and the inclusion of such factors on survey instruments, may facilitate more nuanced understanding of HRQOL.

Although the survey completion rate in our study was high, the fact that approximately 20% of survey opportunities were declined indicates that there may yet be opportunities for improvement of the completion rate, particularly among the most vulnerable patients. Options such as electronic survey administration may increase participation while reducing burden on those administering the survey [25]. Shortening the KDQOL instrument by eliminating less relevant items, and thereby reducing burden on patients and those administering the survey, could also be considered.

This study should be interpreted within the context of its limitations. A major limitation is that this study can, by definition, only report scores and correlations for patients who complete surveys. Patients who decline to complete the survey, or those who are not offered the survey due to clinical or other circumstances, cannot be considered. This may result in under-representation of some of the most vulnerable patients; indeed, previous work has shown that patients who decline to complete HRQOL surveys are at significantly greater risk of mortality [16]. Furthermore, not all patients are eligible to complete the survey: for example, patients who are under the age of 18 or those who have a diagnosis of dementia are excluded. The degree to which the findings of this study extend to these under-represented groups is not known. A minority of patients may receive assistance from social workers or other staff members in completing their KDQOL-36™ surveys. Unfortunately our source data did not permit us to distinguish surveys that were completed with assistance, and thus a sub-group analysis of such surveys was not possible. Similarly, a minority of patients may have switched modalities during the study period, thus contributing surveys to multiple modality groups. However, because approximately 85% of patients are treated with ICHD at any given time, the number of patients who contributed surveys to multiple modalities will necessarily be quite small. Finally, because the intent of this study was to provide a descriptive overview of scores on the KDQOL-36™ among contemporary dialysis patients, no attempts were made to adjust scores for case-mix variables. Rather, the findings presented here are intended to serve as the basis for designing or interpreting future predictive or associative analyses.

## Conclusions

HRQOL is a key outcome for dialysis patients. The KDQOL-36™ is the most widely used tool for assessment of this outcome in the United States. Although the tool is in widespread use and its psychometric properties have been validated, the instrument may not address factors, particularly symptoms, that are the most important to patients in a contemporary setting. New or revised HRQOL assessment tools may be designed to address those factors that are most important to dialysis patients. Improved instruments may in turn provide a more robust foundation to guide interventions aimed at improving HRQOL in patients with ESRD.

## Additional file

Additional file 1: **Table S1.** Characteristics of Unique Patients with Survey Opportunities 2014–2016. **Table S2.** Patient Characteristics in Study Population and United States Renal Data System Population. **Table S3.** Patient Demographics and Clinical Characteristics by KDQOL-36TM Completion Status among Patients on In-Center Hemodialysis. **Table S4.** Patient Demographics and Clinical Characteristics by KDQOL-36TM Completion Status among Patients on Peritoneal Dialysis. **Table S5.** KDQOL-36TM Completion Rates by Patient Characteristics among Patients on In-Center Hemodialysis. **Table S6.** KDQOL-36TM Completion Rates by Patient Characteristics among Patients on Peritoneal Dialysis. **Table S7.** Percent of Surveys with Missing Responses for Items 13–36. **Table S8.** KDQOL-36TM Domain Scores by Calendar Year and Dialysis Vintage at Provider. **Table S9.** KDQOL-TM Scores among Patients Treated with In-Center Hemodialysis by Number of Missed Treatments in the 30 Days Prior to Survey. **Figure S1.** Flow Diagram of Surveys Analyzed. **Figure S2.** Responses to Items on the KDQOL-36TM Symptoms and Problems of Kidney Disease Subscale among Patients on Peritoneal Dialysis. **Figure S3.** Correlation between Selected KDQOL-36TM Items and Domain Scores among Patients on Peritoneal Dialysis. (DOCX 259 kb)

## Abbreviations

BKD: Burden of Kidney Disease
BMI: Body Mass Index
CCI: Charlson Comorbidity Index
CMS: Centers for Medicaid and Medicare Services
EKD: Effects of Kidney Disease
ESRD: End Stage Renal Disease
HHD: Home Hemodialysis
HRQO: Health-Related Quality of Life
ICHD: In-Center Hemodialysis
IDWG: Inter-Dialytic Weight Gain
IRB: Institutional Review Board
KDQOL-36™: Kidney Disease Quality of Life 36
LDO: Large Dialysis Organization
MCS: Mental Component Summary
NOC: Nocturnal Dialysis
PCS: Physical Component Summary
PD: Peritoneal Dialysis
SPKD: Symptoms and Problems of Kidney Disease
