# Correction to: Use of the KDQOL-36™ for assessment of health-related quality of life among dialysis patients in the United States

**DOI:** 10.1186/s12882-019-1630-5

**Published:** 2019-12-10

**Authors:** Dena E. Cohen, Andrew Lee, Scott Sibbel, Deborah Benner, Steven M. Brunelli, Francesca Tentori

**Affiliations:** 1 DaVita Clinical Research, 825 S 8th St, Minneapolis, MN 55404 USA; 2 DaVita, Inc., Denver, CO USA

**Correction to: BMC Nephrol. 2019 Apr 1;20(1):112**

**https://doi.org/10.1186/s12882-019-1295-0**

Following publication of the original article [1], the authors reported an error in Fig. 3 and Additional file 1: Figure S3. This arose due to a coding error during the original analysis. The information in these two figures is supplanted by that provided in Table 4 and Additional file 1: Table S10, below. The correction of this error necessitates three changes to the text of the manuscript.

**Fig. 3:**
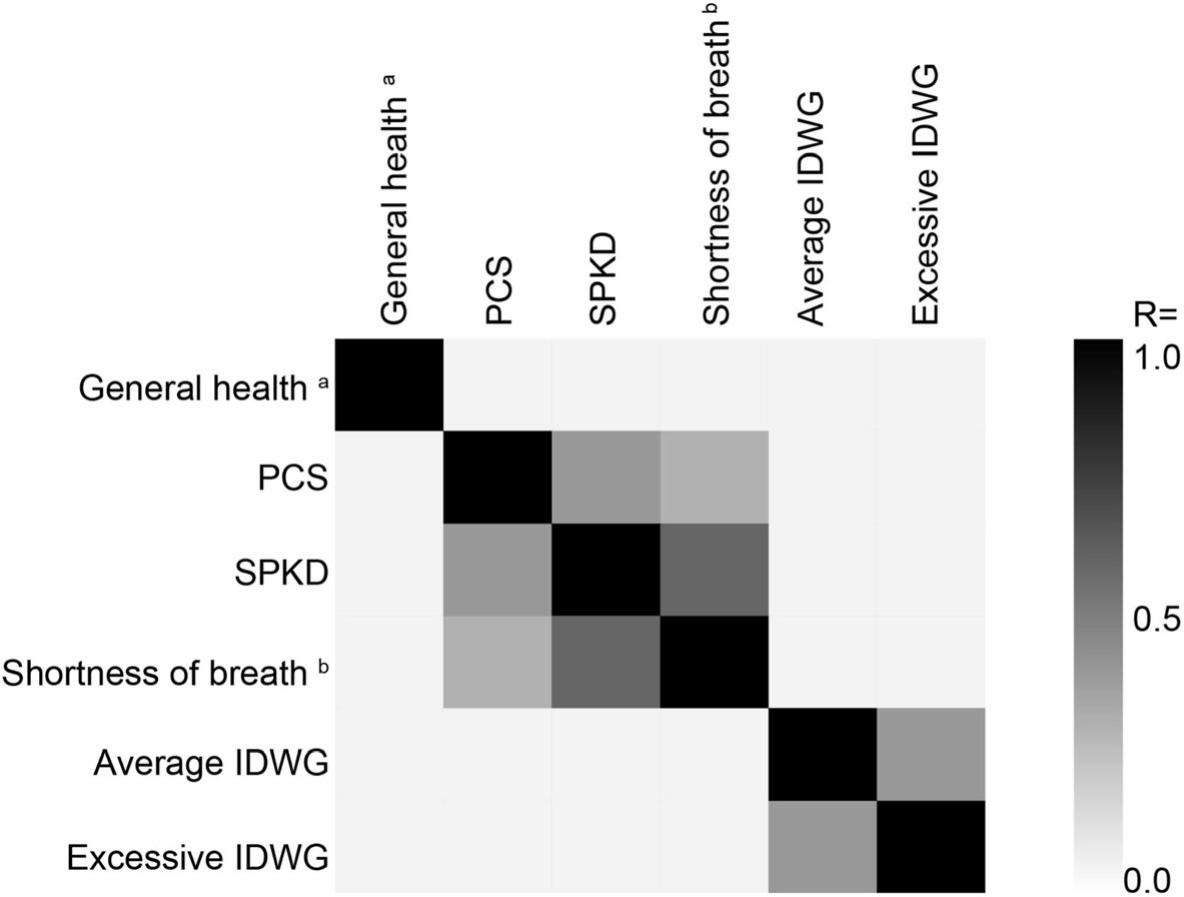
Correlation between Selected KDQOL-36™ Domain Scores, Individual Items, and Indicators of Fluid Overload among Patients on In-Center Hemodialysis. Pearson correlations between the indicated constructs among patients treated with in-center hemodialysis are shown. Average IDWG was considered as a percentage of body weight with respect to treatments in the 30 days prior to survey date. Excessive IDWG was considered as a gain of > 5% of target weight in > 10% of treatments occurring in the 30 days prior to the survey date. ^a^ Item 1: “In general, would you say your health is:” Possible responses are “excellent,” “very good,” “good,” “fair,” and “poor.” ^b^ Item 22: “During the past 4 weeks, to what extent were you bothered by each of the following?” Possible responses are “not at all bothered,” “somewhat bothered,” “moderately bothered,” “very much bothered,” and “extremely bothered.” Abbreviations: *IDWG* interdialytic weight gain, *PCS* physical component summary, *SPKD* symptoms and problems of kidney disease

**Table 4:** Pearson Correlations between Selected KDQOL-36™ Domain Scores, Individual Items, and Indicators of Fluid Overload among Patients on In-Center Hemodialysis (replaces Fig. 3)

	General health^a^	PCS	SPKD	Shortness of breath^b^	Average IDWG	Excessive IDWG
General health^a^	1.000	0.487	0.352	0.263	− 0.021	− 0.022
PCS	0.487	1.000	0.406	0.287	−0.003	−0.006
SPKD	0.352	0.406	1.000	0.601	−0.033	−0.042
Shortness of breath^b^	0.263	0.287	0.601	1.000	−0.015	−0.045
Average IDWG	−0.22	−0.045	− 0.042	−0.045	1.000	0.428
Excessive IDWG	−0.021	−0.006	− 0.059	−0.015	0.428	1.000

Average IDWG was considered as a percentage of body weight with respect to treatments in the 30 days prior to survey date. Excessive IDWG was considered as a gain of > 5% of target weight in > 10% of treatments occurring in the 30 days prior to the survey date

^a^ Item 1: “In general, would you say your health is:” Possible responses are “excellent,” “very good,” “good,” “fair,” and “poor”

^b^ Item 22: “During the past 4 weeks, to what extent were you bothered by each of the following?” Possible responses are “not at all bothered,” “somewhat bothered,” “moderately bothered,” “very much bothered,” and “extremely bothered”

Abbreviations: *IDWG* interdialytic weight gain, *PCS* physical component summary, *SPKD* symptoms and problems of kidney disease

First, in the abstract, it was stated that: “Patient perceptions of general health were not correlated (R<0.05) with PCS or SPKD.” We correct this statement to “Patient perceptions of general health were correlated with PCS and SPKD (R = 0.487 and 0.352, respectively).”

Second, in the results section, it was stated that: “However, patients’ responses to item 1 on the KDQOL-36™ (“In general, would you say your health is:” a component of PCS) did not correlate with either PCS or SPKD (R < 0.05).” We correct this statement to “Patients’ responses to item 1 on the KDQOL-36TM were correlated with both PCS and SPKD (R = 0.487 and 0.352, respectively).”

Third, in the discussion section, it was stated that: “Strikingly, the response to item 1 on the KDQOL-36™ (“In general, would you say your health is”) was not correlated with any of the 5 subscale scores, nor with the response to any individual item on SPKD. This is notable in that patient-reported general health is thought to reflect aspects of health that are difficult to capture via clinical measures, and is independently associated with mortality risk [24]. This finding suggests that efforts to identify factors that influence perceptions of general health among dialysis patients, and the inclusion of such factors on survey instruments, may facilitate more nuanced understanding of HRQOL.” Because these statements are not supported by the corrected analysis, this paragraph should be removed.

## Supplementary information

**Additional file 1: Table S10.** Pearson Correlations between Selected KDQOL-36^TM^ Domain Scores, Individual Items, and Indicators of Fluid Overload among Patients on In-Center Hemodialysis (replaces Figure S3). **Figure S3.** Pearson correlations between the indicated constructs among patients treated with peritoneal dialysis are shown. ^a^ Item 1: “In general, would you say your health is:” Possible responses are “excellent,” “very good,” “good,” “fair,” and “poor.” ^b^ Item 22: “During the past 4 weeks, to what extent were you bothered by each of the following?” Possible responses are “not at all bothered,” “somewhat bothered,” “moderately bothered,” “very much bothered,” and “extremely bothered.” Abbreviations: *PCS* physical component score, *SPKD* symptoms and problems of kidney disease.
