# Supplementary material for: Use of the KDQOL-36™ for assessment of health-related quality of life among dialysis patients in the United States
Source: BMC Nephrol. 2019 Apr 1;20:112. doi: 10.1186/s12882-019-1295-0 (PMC6444438; doi:10.1186/s12882-019-1295-0)
Supplement: Supplementary file 1 — Table S1. Characteristics of Unique Patients with Survey Opportunities 2014–2016. Table S2. Patient Characteristics in Study Population and United States Renal Data System Population. Table S3. Patient Demographics and Clinical Characteristics by KDQOL-36TM Completion Status among Patients on In-Center Hemodialysis. Table S4. Patient Demographics and Clinical Characteristics by KDQOL-36TM Completion Status among Patients on Peritoneal Dialysis. Table S5. KDQOL-36TM Completion Rates by Patient Characteristics among Patients on In-Center Hemodialysis. Table S6. KDQOL-36TM Completion Rates by Patient Characteristics among Patients on Peritoneal Dialysis. Table S7. Percent of Surveys with Missing Responses for Items 13–36. Table S8. KDQOL-36TM Domain Scores by Calendar Year and Dialysis Vintage at Provider. Table S9. KDQOL-TM Scores among Patients Treated with In-Center Hemodialysis by Number of Missed Treatments in the 30 Days Prior to Survey. Figure S1. Flow Diagram of Surveys Analyzed. Figure S2. Responses to Items on the KDQOL-36TM Symptoms and Problems of Kidney Disease Subscale among Patients on Peritoneal Dialysis. Figure S3. Correlation between Selected KDQOL-36TM Items and Domain Scores among Patients on Peritoneal Dialysis. (DOCX 259 kb) [file 12882_2019_1295_MOESM1_ESM.docx]

**Supplemental Information**

**Table S1: Characteristics of Unique Patients with Survey Opportunities 2014-2016**

|  | **Patients with Survey Opportunities 2014-2016**  **N=240,343** |
| --- | --- |
| **Age,** years, mean ± SD | 61.3 ± 15.0 |
| **Sex**, female, n (%) | 134,088 (55.8) |
| **Race**, n (%) |  |
| White | 95,903 (39.9) |
| Black | 82,308 (34.3) |
| Hispanic | 41,782 (17.4) |
| Asian | 10,047 (4.2) |
| Other/unknown | 10,303 (4.3) |
| **Modality**, n (%) |  |
| ICHD | 208,236 (86.6) |
| PD | 26,843 (11.2) |
| HHD | 3611 (1.5) |
| NOC | 1644 (0.7) |
| **Dialysis vintage,** months, median [p25, p75] | 18 [4,53] |
| **BMI**, kg/m^2^, mean ± SD | 28.4 ± 7.3 |
| **CCI**, median [p25, p75] | 5.3 ± 1.9 |
| **Diabetes**, n (%) | 162,546 (67.6) |
| All characteristics were assessed as of the patient’s first survey opportunity during the study period.  Abbreviations: BMI, body mass index; CCI, Charlson comorbidity index; HHD, home hemodialysis; ICHD, in-center hemodialysis; NOC, nocturnal dialysis; PD, peritoneal dialysis; SD, standard deviation | |

**Table S2: Patient Characteristics in Study Population and United States Renal Data System Population**

|  | **2017 USRDS ^a^** | **Current Study Population** |
| --- | --- | --- |
| **Age** ^b^ |  |  |
| 22-44* | 12.2 | 12.8 |
| 45-64 | 41.7 | 42.0 |
| 65+ | 45.5 | 44.2 |
| **Sex** |  |  |
| Male | 57.0 | 55.6 |
| Female | 43.0 | 44.4 |
| **Race ^c^** |  |  |
| White | 57.5 | 38.8 |
| Black/African American | 35.1 | 35.2 |
| American Indian or Alaska Native | 1.1 | Data not available |
| Asian | 4.5 | 4.2 |
| Native Hawaiian or Pacific Islander | 1.4 | Data not available |
| Other or Multiracial | 0.3 | Data not available |
| Unknown | 0.1 | 4.2 |
| **Ethnicity ^c^** |  |  |
| Hispanic | 18.3 | 17.6 |
| Non-Hispanic | 81.2 | Data not available |
| Unknown | 0.5 | Data not available |
| **Diabetes ^d^** | 44.9 | 68.5 |
| **Modality ^e^** |  |  |
| Hemodialysis | 90.0 | 88.9 |
| Peritoneal dialysis | 10.0 | 11.1 |
| ^a^ Data derived from Table 1.6 of USRDS 2017 data report, reflecting patients dialyzing in CY 2015. Only prevalent patients on HD and PD were considered in order to ensure fair comparisons with the population analyzed in the current report.  ^b^ Age categories used by USRDS and within the current study did not align perfectly; the category listed as 22-44 represents ages 22-44 in the USRDS 2017 data report and ages 25-44 in the current study population.  ^c^ Race categories used by USRDS and within the current study did not align perfectly. In the current study population, white race and Hispanic ethnicity were treated as mutually exclusive categories, which was not the case for USRDS. USRDS also reports race categories not captured in the data for the current study population; these other categories are likely represented in the “Unknown” category for the current study.  ^d^ In the USRDS data report, diabetes is included only as a primary cause of end-stage renal disease, whereas it is included as a comorbidity in the data for the current study population, likely explaining why the reported prevalence is higher in this group.  ^e^ Hemodialysis is reported as a single category in the USRDS data report. In the current study population, the “hemodialysis” category includes in-center hemodialysis, home hemodialysis, and nocturnal dialysis, which were analyzed separately elsewhere in the manuscript. | | |

**Table S3: Patient Demographics and Clinical Characteristics by KDQOL-36^TM^ Completion Status among Patients on In-Center Hemodialysis**

|  | **Overall**  **N = 358,475** | **Declined**  **N = 75,580** | **Completed**  **N= 282,895** |
| --- | --- | --- | --- |
| **Age,** years, mean ± SD | 62.1 ± 14.8 | 64.7 ± 15.1 | 61.4 ± 14.6 |
| **Sex**, female, n (%) | 159,434 (44.5) | 32,955 (43.6) | 126,479 (44.7) |
| **Race**, n (%) |  |  |  |
| White | 132,805 (37.4) | 26,588 (35.2) | 106,217 (37.5) |
| Black | 130,553 (36.4) | 25,633 (33.9) | 104,920 (37.1) |
| Hispanic | 64,943 (18.1) | 13,771 (18.2) | 51,172 (18.1) |
| Asian | 14,687 (4.1) | 5375 (7.1) | 9312 (3.3) |
| Other/unknown | 15,487 (4.3) | 4213 (5.6) | 11,274 (4.0) |
| **Vascular access**, n (%) |  |  |  |
| Arteriovenous fistula | 224,186 (62.5) | 45,307 (59.9) | 178,879 (63.2) |
| Arteriovenous graft | 62,158 (17.3) | 13,945 (18.5) | 48,213 (17.0) |
| Central venous catheter | 72,125 (20.1) | 16,328 (21.6) | 55,783 (19.7) |
| **Dialysis vintage,** months, median [p25, p75] | 30 [13, 59] | 36 [15, 64] | 29 [12, 57] |
| **BMI**, kg/m^2^, mean ± SD | 28.4 ± 7.4 | 27.0 ± 6.9 | 28.8 ± 7.5 |
| **CCI**, median [p25, p75] | 6 [4, 7] | 6 [4, 7] | 5 [6, 7] |
| **Diabetes**, n (%) | 249,163 (69.5) | 53,436 (70.7) | 195,727 (69.2) |
| Abbreviations: BMI, body mass index; CCI, Charlson comorbidity index; SD, standard deviation | | | |

**Table S4: Patient Demographics and Clinical Characteristics by KDQOL-36^TM^ Completion Status among Patients on Peritoneal Dialysis**

|  | **Overall**  **N = 45,903** | **Declined**  **N = 6395** | **Completed**  **N=39,508** |
| --- | --- | --- | --- |
| **Age,** years, mean ± SD | 57.5 ± 15.2 | 58.9 ± 15.8 | 57.2 ± 15.1 |
| **Sex**, female, n (%) | 20,793 (45.3) | 2850 (44.6) | 17,943 (45.4) |
| **Race**, n (%) |  |  |  |
| White | 22,682 (49.4) | 3171 (49.6) | 19,511 (49.3) |
| Black | 12,181 (26.5) | 1475 (23.1) | 10,706 (27.1) |
| Hispanic | 6847 (14.9) | 910 (14.2) | 5937 (15.0) |
| Asian | 2474 (5.4) | 534 (8.4) | 1940 (4.9) |
| Other/unknown | 1719 (3.7) | 305 (4.8) | 1414 (3.6) |
| **Dialysis vintage,** months, median [p25, p75] | 21 [7, 41] | 25 [11, 46] | 20 [6, 40] |
| **BMI**, kg/m^2^, mean ± SD | 29.3 ± 6.7 | 28.5 ± 6.7 | 29.4 ± 6.7 |
| **CCI**, median [p25, p75] | 5 [4, 6] | 5 [4, 6] | 5 [4, 6] |
| **Diabetes**, n (%) | 28,760 (62.7) | 4059 (63.5) | 24,701 (62.5) |
| Abbreviations: BMI, body mass index; CCI, Charlson comorbidity index; SD, standard deviation | | | |

**Table S5: KDQOL-36^TM^ Completion Rates by Patient Characteristics among Patients on In-Center Hemodialysis**

|  | **Declined** | **Completed** |
| --- | --- | --- |
| **All** | 75,580 (21.1) | 282,895 (78.9) |
| **Age**, years |  |  |
| 18-24 | 535 (16.8) | 2642 (83.2) |
| 25-34 | 2507 (18.6) | 10,967 (81.4) |
| 35-44 | 4810 (17.0) | 23,556 (83.0) |
| 45-54 | 10,101 (17.5) | 47,507 (82.5) |
| 55-64 | 17,190 (18.9) | 73,683 (81.1) |
| 65+ | 40,437 (24.5) | 124,540 (75.5) |
| **Sex** |  |  |
| Male | 42,625 (21.4) | 156,416 (78.6) |
| Female | 32,955 (20.7) | 126,479 (79.3) |
| **Race/Ethnicity** |  |  |
| White | 26,558 (20.0) | 106,217 (80.0) |
| Black | 25,633 (19.6) | 104,920 (80.4) |
| Hispanic | 13,771 (21.2) | 51,172 (78.8) |
| Asian | 5375 (36.6) | 9312 (63.4) |
| Other/Unknown/Missing | 4213 (27.2) | 11,274 (72.8) |
| **Vintage**, months |  |  |
| <12 | 17,155 (18.5) | 75,436 (81.5) |
| 12-24 | 11,459 (19.1) | 48,443 (80.9) |
| 25-36 | 9835 (20.5) | 38,145 (79.5) |
| 36+ | 37,131 (23.5) | 120,871 (76.5) |
| **BMI** |  |  |
| <18.5 | 4489 (32.3) | 9421 (67.7) |
| 18.5-24 | 29,781 (25.2) | 88,393 (74.8) |
| 25-29 | 21,194 (20.6) | 81,675 (79.4) |
| 30+ | 20,116 (16.3) | 103406 (83.7) |
| **CCI** |  |  |
| <3 | 4461 (17.4) | 21,150 (82.6) |
| 3-4 | 14,774 (17.5) | 69,616 (82.5) |
| 5-6 | 29,248 (20.2) | 115,590 (79.8) |
| 7+ | 27,097 (26.1) | 76,539 (73.9) |
| **Vascular Access** |  |  |
| Arteriovenous fistula | 45,307 (20.2) | 178,879 (79.8) |
| Arteriovenous graft | 13,945 (22.4) | 48,213 (77.6) |
| Central venous catheter | 16,328 (22.6) | 55,803 (77.4) |
| **Missed Tx due to Hospitalization ^a^** |  |  |
| 0 | 72,036 (20.8) | 274,146 (79.2) |
| 1 | 1001 (24.3) | 3112 (75.7) |
| 2 | 777 (26.3) | 2177 (73.7) |
| 3+ | 1766 (33.8) | 3460 (66.2) |
| **Missed Tx due to Absence ^a^** |  |  |
| 0 | 70,077 (21.1) | 262,283 (78.9) |
| 1 | 2980 (20.0) | 11,893 (80.0) |
| 2 | 1122 (20.9) | 4253 (79.1) |
| 3+ | 1401 (23.9) | 4466 (76.1) |
| ^a^ During the 30 days prior to the survey opportunity  Abbreviations: BMI, body mass index; CCI, Charlson comorbidity index; SD, standard deviation; Tx, treatment | | |

**Table S6: KDQOL-36^TM^ Completion Rates by Patient Characteristics among Patients on Peritoneal Dialysis**

|  | **Declined** | **Completed** |
| --- | --- | --- |
| **All** | 6395 (13.9) | 39,508 (86.1) |
| **Age**, years |  |  |
| 18-24 | 136 (15.0) | 768 (85.0) |
| 25-34 | 409 (13.4) | 2648 (86.6) |
| 35-44 | 692 (12.4) | 4877 (87.6) |
| 45-54 | 1102 (12.5) | 7716 (87.5) |
| 55-64 | 1513 (13.2) | 9917 (86.8) |
| 65+ | 2543 (15.8) | 13,582 (84.2) |
| **Sex** |  |  |
| Male | 3545 (14.1) | 21,565 (85.9) |
| Female | 2850 (13.7) | 17,943 (86.3) |
| **Race/Ethnicity** |  |  |
| White | 3171 (14) | 19,511 (86.0) |
| Black | 1475 (12.1) | 107,06 (87.9) |
| Hispanic | 910 (13.3) | 5937 (86.7) |
| Asian | 534 (21.6) | 1940 (78.4) |
| Other/Unknown/Missing | 305 (17.7) | 1414 (82.3) |
| **Vintage**, months |  |  |
| <12 | 1835 (11.9) | 13,533 (88.1) |
| 12-24 | 1374 (13.5) | 8790 (86.5) |
| 25-36 | 1000 (14.4) | 5949 (85.6) |
| 36+ | 2186 (16.3) | 11,236 (83.7) |
| **BMI** |  |  |
| <18.5 | 612 (22.1) | 2162 (77.9) |
| 18.5-24 | 1851 (15.8) | 9863 (84.2) |
| 25-29 | 1806 (13.2) | 11,851 (86.8) |
| 30+ | 2126 (12.0) | 15,632 (88.0) |
| **CCI** |  |  |
| <3 | 735 (12.6) | 5097 (87.4) |
| 3-4 | 1738 (12.5) | 12,144 (87.5) |
| 5-6 | 2377 (13.8) | 14,840 (86.2) |
| 7+ | 1545 (17.2) | 7427 (82.8) |
| Abbreviations: BMI, body mass index; CCI, Charlson comorbidity index; SD, standard deviation | | |

**Table S7: Percent of Surveys with Missing Responses for Items 13-36**

|  | **Missingness among Surveys**  **with Items 1-12 Complete**  **N=330,412** |
| --- | --- |
| **Item number,** missingness, n (%) |  |
| 13 | 1582 (0.5) |
| 14 | 1571 (0.5) |
| 15 | 1545 (0.5) |
| 16 | 1640 (0.5) |
| 17 | 2658 (0.8) |
| 18 | 2539 (0.8) |
| 19 | 2340 (0.7) |
| 20 | 2674 (0.8) |
| 21 | 2435 (0.7) |
| 22 | 2147 (0.7) |
| 23 | 2092 (0.6) |
| 24 | 2428 (0.7) |
| 25 | 2537 (0.8) |
| 26 | 2381 (0.7) |
| 27 | 2299 (0.7) |
| 28 ^a^ | 11,019 (3.3) |
| 29 | 2643 (0.8) |
| 30 | 2726 (0.8) |
| 31 | 2891 (0.9) |
| 32 | 3289 (1.0) |
| 33 | 2937 (0.9) |
| 34 | 2863 (0.9) |
| 35 ^b^ | 18,970 (5.7) |
| 36 | 4217 (1.3) |
| ^a^ (Hemodialysis patient only) Problems with your access site?  (Peritoneal dialysis patient only) Problems with your catheter site?  ^b^ Your sex life? | |

**Table S8: KDQOL-36^TM^ Domain Scores by Calendar Year and Dialysis Vintage at Provider**

|  | **Survey Opportunities**  n (%) | | **KDQOL-36^TM^ Domains**  mean ± SD | | | | |
| --- | --- | --- | --- | --- | --- | --- | --- |
|  | **Declined** | **Completed** | **PCS** | **MCS** | **BKD** | **SPKD** | **EKD** |
| **Calendar Year** | | | | | | | |
| 2014 | 31,809 (22.0) | 112,790 (78.0) | 36.3 ± 12.2 | 48.6 ± 13.5 | 51.0 ± 29.7 | 77.5 ± 16.8 | 72.0 ± 22.7 |
| 2015 | 26,935 (19.7) | 110,099 (80.3) | 36.6 ± 12.2 | 48.9 ± 13.4 | 51.2 ± 29.8 | 78.0 ± 16.8 | 72.9 ± 22.7 |
| 2016 | 24,808 (18.7) | 107,523 (81.3) | 36.9 ± 12.2 | 49.5 ± 13.3 | 51.6 ± 30.0 | 78.8 ± 16.7 | 74.0 ± 22.5 |
| **Survey Window** | | | | | | | |
| 4 mo | 10579 (16.3) | 54331 (83.7) | 36.2 ± 12.0 | 49.0 ± 13.4 | 49.6 ± 29.3 | 78.6 ± 16.2 | 72.8 ± 22.3 |
| 16 mo | 16,565 (18.8) | 71,603 (81.2) | 36.5 ± 12.3 | 48.8 ± 13.5 | 49.9 ± 29.6 | 77.9 ± 16.8 | 72.6 ± 22.6 |
| 28 mo | 13,988 (19.7) | 57,089 (80.3) | 36.8 ± 12.3 | 49.1 ± 13.3 | 51.0 ± 29.7 | 78.0 ± 16.9 | 72.9 ± 22.7 |
| Abbreviations: BKD, burden of kidney disease; EKD, effects of kidney disease; MCS, mental component score; PCS, physical component score; SPKD, symptoms and problems of kidney disease. | | | | | | | |

**Table S9: KDQOL-TM Scores among Patients Treated with In-Center Hemodialysis by Number of Missed Treatments in the 30 Days Prior to Survey**

|  | **PCS** | **MCS** | **BKD** | **SPKD** | **EKD** |
| --- | --- | --- | --- | --- | --- |
| Missed Treatment due to Hospitalization | | | | | |
| **0** | 36.7 ± 12.2 | 49.1 ± 13.4 | 51.4 ± 29.8 | 78.2 ± 16.7 | 73.0 ± 22.6 |
| **1** | 33.7 ± 11.7 | 47.5 ± 13.9 | 46.6 ± 30.3 | 74.5 ± 18.4 | 71.4 ± 23.8 |
| **2** | 33.2 ± 10.9 | 47.7 ± 13.1 | 46.4 ± 29.7 | 74.5 ± 17.8 | 70.7 ± 23.2 |
| **3+** | 31.5 ± 11.0 | 46.6 ± 13.8 | 45.5 ± 30.1 | 74.1 ± 17.9 | 70.4 ± 24.1 |
| Missed Treatment due to Other Reasons ^a^ | | | | | |
| **0** | 36.6 ± 12.2 | 49.1 ± 13.4 | 51.5 ± 29.8 | 78.3 ± 16.7 | 73.0 ± 22.6 |
| **1** | 36.1 ± 12.1 | 48.5 ± 13.5 | 49.0 ± 30.0 | 76.8 ± 17.4 | 73.0 ± 22.9 |
| **2** | 35.7 ± 12.2 | 47.3 ± 13.7 | 47.3 ± 30.1 | 75.7 ± 17.9 | 72.7 ± 23.2 |
| **3+** | 35.8 ± 12.0 | 46.6 ± 13.7 | 45.1 ± 29.6 | 74.2 ± 18.6 | 71.7 ± 23.6 |
| ^a^ Represents missed treatments for which no medical reason (hospitalization, emergency department visit) was recorded  Abbreviations: BKD, burden of kidney disease; EKD, effects of kidney disease; HHD, home hemodialysis; ICHD, in-center hemodialysis; MCS, mental component score; NOC, nocturnal dialysis; PCS, physical component score; PD, peritoneal dialysis; SPKD, symptoms and problems of kidney disease. | | | | | |

**Figure S1: Flow Diagram of Surveys Analyzed**

**
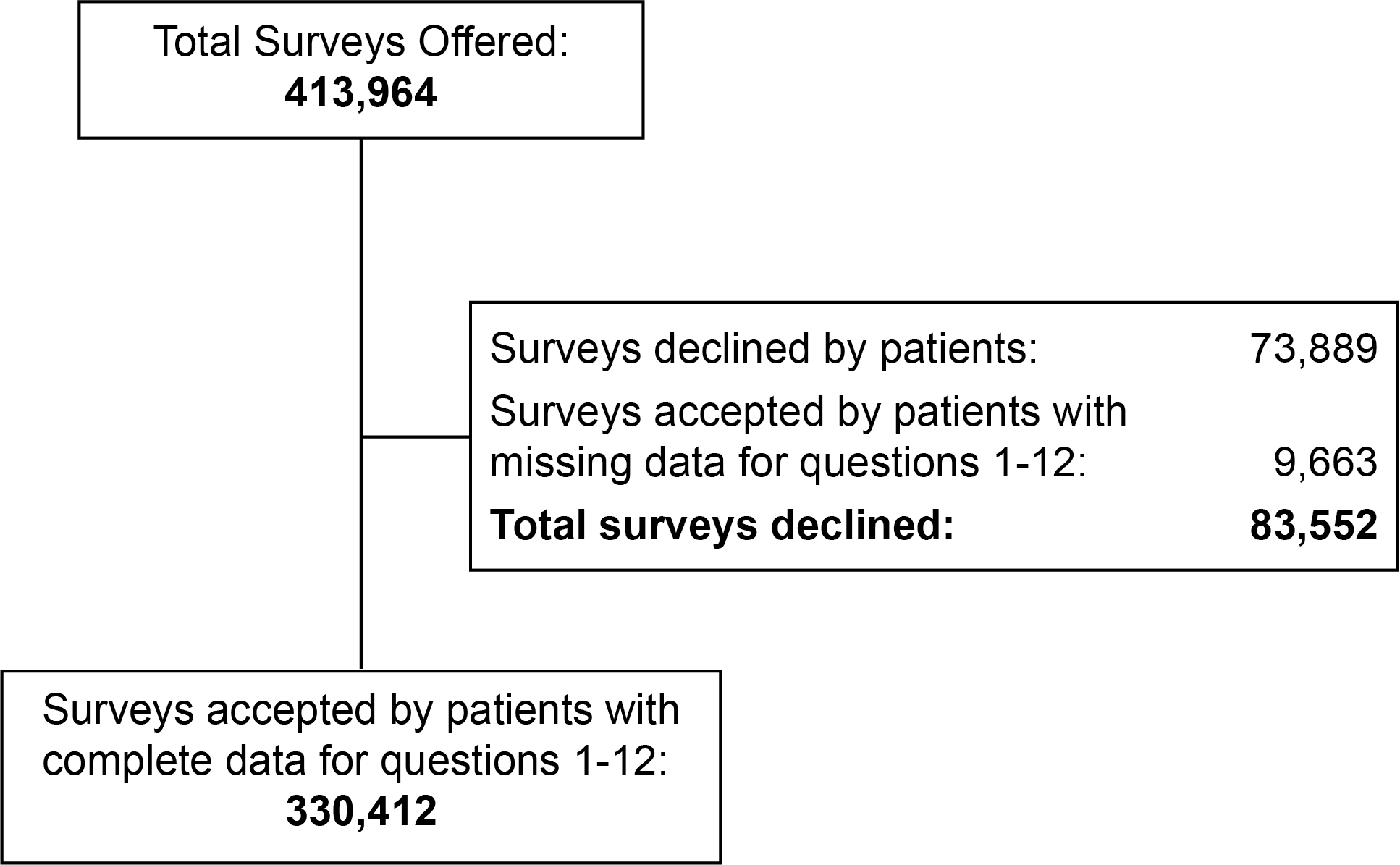
**

A total of 413,964 KDQOL-36^TM^ suveys were offered to patients dialyzing at the LDO 2014-2016. Of these, 73,889 surveys were declined by patients at the time of offering. A further 9663 surveys were accepted by patients at the time of offering; however, responses to one or more of Items 1-12 were missing, and thus the surveys were counted as declined, for a total of 83,552 declined surveys. This left a remainder of 330,412 surveys completed surveys during the study period.

**Figure S2: Responses to Items on the KDQOL-36^TM^ Symptoms and Problems of Kidney Disease Subscale among Patients on Peritoneal Dialysis**


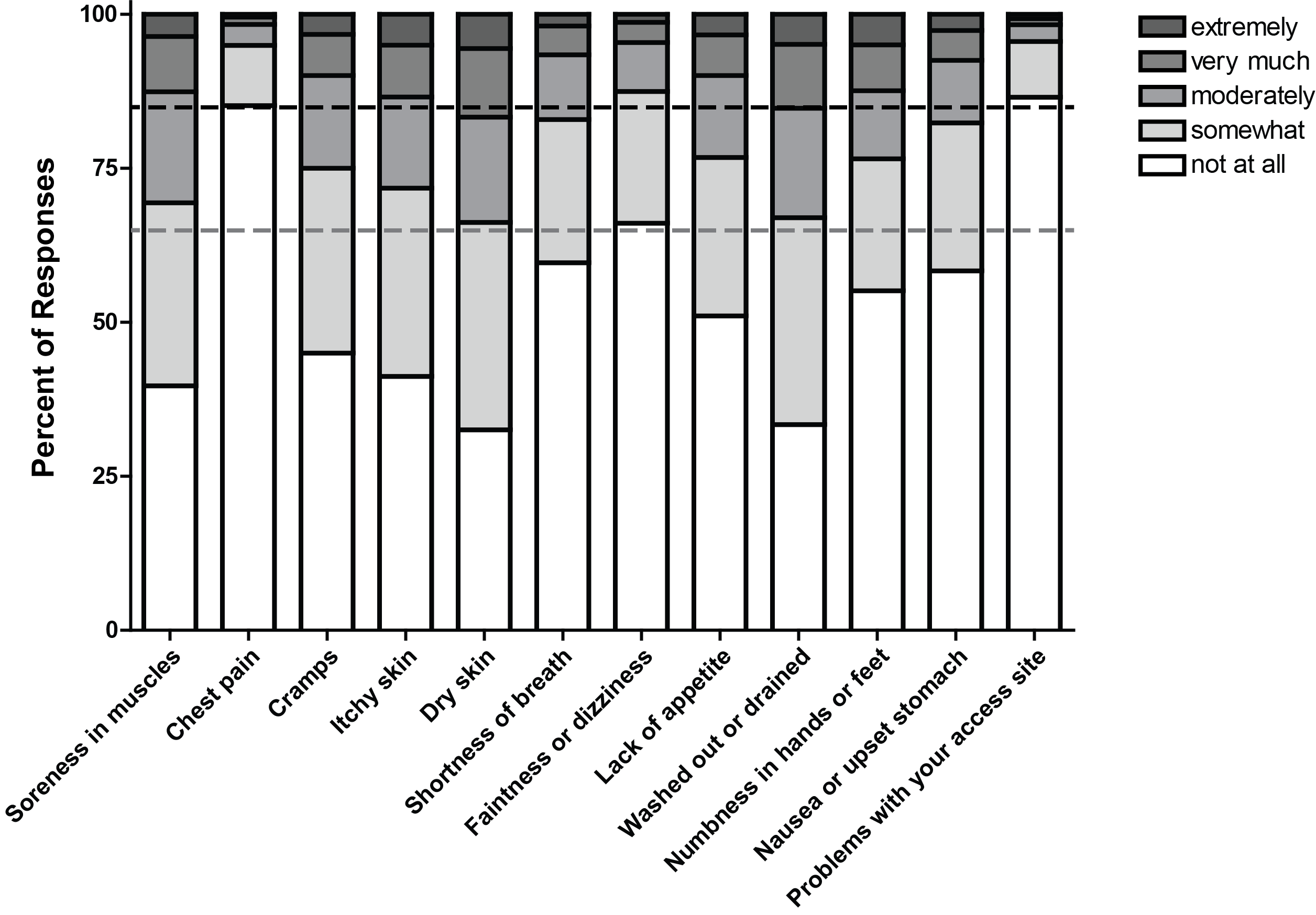


Responses to the 12 items comprising the Symptoms and Problems of Kidney Disease subscale among patients treated with peritoneal dialysis who responded to each item are depicted. The question stem for all 12 items is, “During the past 4 weeks, to what extent were you bothered by each of the following?” Possible responses are not at all, somewhat, moderately, very much, and extremely. Dashed grey line indicates cumulative 65% of responses; dashed black line indicates cumulative 85% of responses.

**Figure S3: Correlation between Selected KDQOL-36^TM^ Items and Domain Scores among Patients on Peritoneal Dialysis**


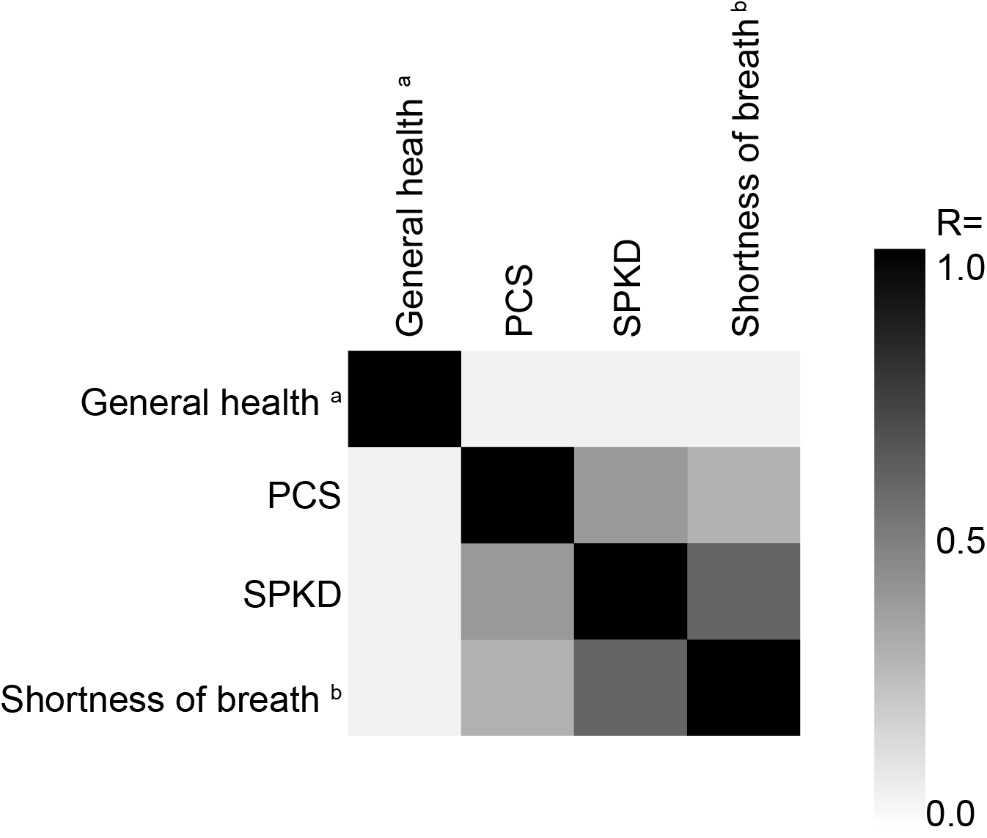


Pearson correlations between the indicated constructs among patients treated with peritoneal dialysis are shown.

^a^ Item 1: “In general, would you say your health is:” Possible responses are “excellent,” “very good,” “good,” “fair,” and “poor.”

^b^ Item 22: “During the past 4 weeks, to what extent were you bothered by each of the following?” Possible responses are “not at all bothered,” “somewhat bothered,” “moderately bothered,” “very much bothered,” and “extremely bothered.”

Abbreviations: PCS, physical component score; SPKD, symptoms and problems of kidney disease
