# Supplementary material for: Correction to: Use of the KDQOL-36™ for assessment of health-related quality of life among dialysis patients in the United States
Source: BMC Nephrol. 2019 Dec 10;20:461. doi: 10.1186/s12882-019-1630-5 (PMC6902527; doi:10.1186/s12882-019-1630-5)

Table S10

|  | **General health^a^** | **PCS** | **SPKD** | **Shortness of breath^b^** |
| --- | --- | --- | --- | --- |
| **General health^a^** | 1.000 | 0.500 | 0.375 | 0.258 |
| **PCS** | 0.500 | 1.000 | 0.430 | 0.300 |
| **SPKD** | 0.375 | 0.430 | 1.000 | 0.578 |
| **Shortness of breath^b^** | 0.258 | 0.300 | 0.578 | 1.000 |
| ^a^ Item 1: “In general, would you say your health is:” Possible responses are “excellent,” “very good,” “good,” “fair,” and “poor.”  ^b^ Item 22: “During the past 4 weeks, to what extent were you bothered by each of the following?” Possible responses are “not at all bothered,” “somewhat bothered,” “moderately bothered,” “very much bothered,” and “extremely bothered.”  Abbreviations: PCS, physical component summary; SPKD, symptoms and problems of kidney disease. | | | | |

Figure S3


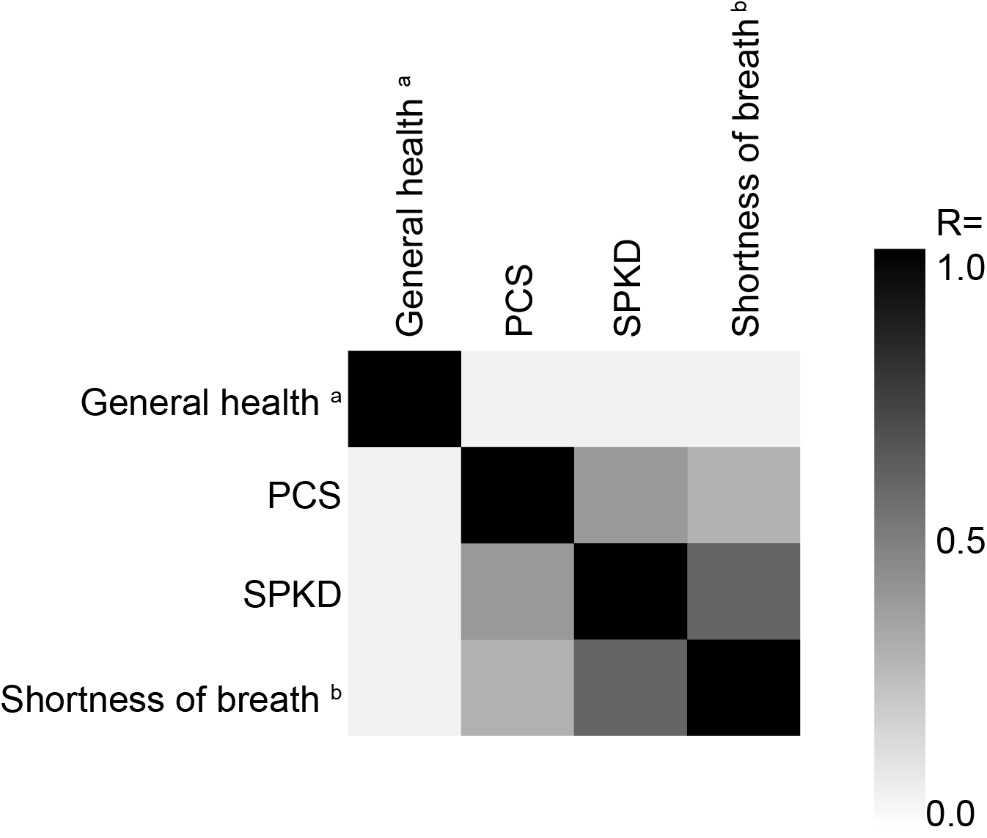

Supplement: Supplementary file 1 — Additional file 1: Table S10. Pearson Correlations between Selected KDQOL-36TM Domain Scores, Individual Items, and Indicators of Fluid Overload among Patients on In-Center Hemodialysis (replaces Figure S3). Figure S3. Pearson correlations between the indicated constructs among patients treated with peritoneal dialysis are shown. a Item 1: “In general, would you say your health is:” Possible responses are “excellent,” “very good,” “good,” “fair,” and “poor.” b Item 22: “During the past 4 weeks, to what extent were you bothered by each of the following?” Possible responses are “not at all bothered,” “somewhat bothered,” “moderately bothered,” “very much bothered,” and “extremely bothered.” Abbreviations: PCS physical component score, SPKD symptoms and problems of kidney disease. [file 12882_2019_1630_MOESM1_ESM.docx]
